# Epidemiology, pathogenesis, clinical presentation and management of TB in patients with HIV and diabetes

**DOI:** 10.5588/ijtld.22.0685

**Published:** 2023-04-01

**Authors:** D. Goletti, R. Pisapia, F. M. Fusco, A. Aiello, R. Van Crevel

**Affiliations:** 1 Translational Research Unit, National Institute for Infectious Diseases Lazzaro Spallanzani-IRCCS, Rome, Italy; 2 Ospedale Cotugno, Azienda Ospedaliera dei Colli, Naples, Italy; 3 Department of Internal Medicine and Radboud Center for Infectious Diseases (RCI), Radboud University Medical Center, Nijmegen, the Netherlands

**Keywords:** tuberculosis, DM, HIV, clinical management, TB-HIV, TB-DM, pathogenesis

## Abstract

Caused by *Mycobacterium tuberculosis*,TBistheleading cause of death from an infectious disease. HIVand diabetes are recognised risk factors for progression of TB disease and both have a strong impact on the diagnosis and management of TB, threatening efforts to end TB globally. Here we provide the latest data on the complex interplay between these conditions. TB patients with HIV present systemic immune activation, increased HIV viral load, more severe clinical presentations and reduced success of TB therapy. Similarly, TB patients with diabetes are characterised by an exaggerated adaptive immunity, worsening of the clinical presentations and a higher risk for multidrug resistance and treatment failure. It is important to strengthen resources to prevent these comorbidities from occurring and to implement screening, early diagnosis and appropriate management strategies.

## Abstract (fr)

Due à *Mycobacterium tuberculosis*, la TB est la première cause de décès par maladie infectieuse. Le VIH et le diabète sont des facteurs de risque reconnus de progression de la TB active et ont tous deux un impact important sur le diagnostic et la prise en charge de la TB, menaçant ainsi les efforts visant à éradiquer la TB dans le monde. Nous présentons ici les dernières données sur l’interaction complexe entre ces maladies. Les patients atteints de TB et infectés par le VIH sont associés à une activation immunitaire systémique, à une augmentation de la charge virale du VIH, à des présentations cliniques plus graves et à une moindre chance de réussite du traitement antituberculeux. De même, les patients atteints de TB et de diabète sont caractérisés par une immunité adaptative exagérée, une aggravation des présentations cliniques et un risque accru de multirésistance et d’échec thérapeutique. Il est important de renforcer les ressources afin de prévenir l’apparition de ces comorbidités et de mettre en œuvre des stratégies de dépistage, de diagnostic précoce et deprise en charge appropriée.

In 2021, an estimated 10.6 million people had TB worldwide, leading to 1.6 million deaths. TB is present in all countries and age groups: 6 million men, 3.4 million women and 1.2 million children. These figures are dramatic, given that TB is curable and preventable. TB is an ongoing pandemic, with increased new cases and deaths in recent years due to the reduction in TB services during the COVID-19 pandemic.^1^ In infected patients, *Mycobacterium tuberculosis* (*Mtb*) replication is controlled through the immune response involving both innate (mostly at the beginning) and adaptive immunity, with important roles for CD4 and CD8 T-cells, although the correlates of protective immunity are not yet clear.^2^ In people at risk of TB, the WHO recommends preventive therapy to reduce the risk of progression to TB disease.^3^ The main five risk factors for progression to TB disease are undernourishment, HIV infection, alcohol use disorders, smoking (especially among men) and diabetes mellitus (DM).^3^ In this review, we focus on the epidemiology, pathogenesis, clinical presentation, treatment and prevention of TB coinfection with HIV and with DM.

## EPIDEMIOLOGY

Globally, there are an estimated 38.4 million people living with HIV (PLWH), and 537 million with DM, mostly type 2 DM.^4^ In 2022, the global estimate of HIV-coinfected patients with TB was 710,000 (6.7% of all TB cases), and 187,000 deaths.^5^ With a pooled DM prevalence of 15.3% in TB patients,^6^ it is likely that at least 1.0–1.5 million people have combined TB-DM. However, numbers are likely to grow with a global doubling of DM prevalence over the next 30 years, the largest increase occurring in sub-Saharan Africa and Asia.

The risk of TB is much higher in PLWH than in those with DM, and strongly associated with the level of immunodeficiency (Figure 1A and B; Table 1). Soon after HIV infection, the risk of TB disease increases 2–5-fold compared to non-HIV-infected individuals. With progression to HIV-induced severe immunodeficiency, the risk of TB is further increased at least 20-fold greater than in the general population. Antiretroviral therapy (ART) for HIV-1 does not fully restore the baseline level of risk.^22^

**Figure:**
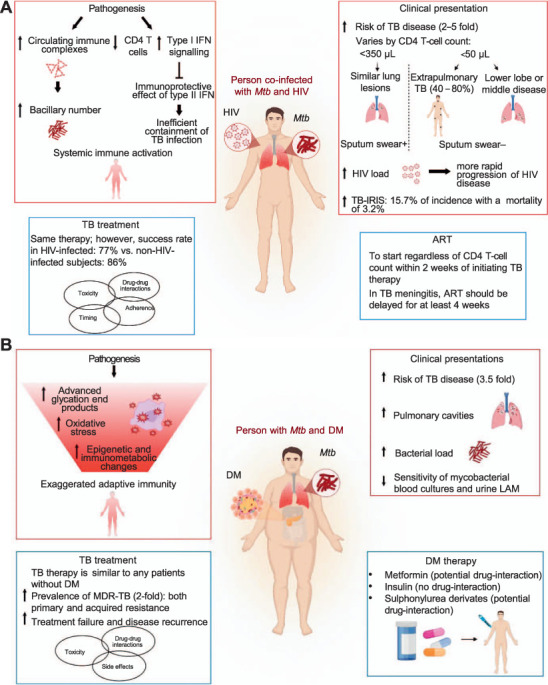
Schematic overview of the pathogenesis, clinical presentations and treatments in subjects with TB and HIV or DM. A) Subjects co-infected with Mtb and HIV are characterised by a systemic immune activation and show an increased risk for TB disease, HIV viral load and incidence of TB-IRIS. The appearance and location of pulmonary lesions vary according to the CD4 T-cell count. Treatment of TB-HIV co-infected individuals has a reduced success rate. ART should be started regardless of CD4 T-cell count and delayed in case of TB meningitis. B) TB-DM patients exhibit an enhanced adaptive immune response compared to those without DM, as well as an increased risk for TB, number and size of pulmonary cavities, and bacterial load. TB patients with DM have a higher risk for multidrug resistance and treatment failure. IFN = interferon; Mtb = M. tuberculosis; IRIS = immune reconstitution inflammatory syndrome; ART =antiretroviral therapy; LAM = lipoarabinomannan; DM = diabetes mellitus; MDR-TB = multidrug-resistant TB.

**Table 1:** Epidemiology, pathogenesis, clinical presentation, treatment and prevention data in TB patients with HIV or DM

	HIV	DM
Global number of estimated cases	38.4 million^5^	420 million^7^
Global number of estimated TB cases in 2021	710,000^8^	1.5 million 9
Relative risk for TB infection	2–5	1.6^10^
Relative risk for TB disease	20^11^	3.59^12^,^13^
Estimated prevalence among TB patients in 2021	6.7%	15%^6^
Diagnosis	In addition to standard TB diagnostic procedures, consider mycobacterial blood culture and urine LAM^14^	TB standard diagnosis procedures
Pulmonary TB	Sometimes atypical, fewer cavities, especially in PLWH with low CD4 counts^15^	Often more cavities^16^
Extrapulmonary/disseminated TB	Increased risk (especially with low CD4)	No increased risk^16^
MDR-TB	1.42-fold higher risk of MDR-TB^17^	2-fold higher risk of MDR-TB^18^
Treatment success	Failure (up to 50%) and high mortality (up to 70%) before the new regimens were in place^8^	Risk ratio for death 1.88 and release 1.64^19^
Issues related to combined treatment	Drug interactions, IRIS	TB drug dosing with higher weight
	Overlapping toxicity	Drug interactions
		Drug toxicity
BCG vaccination	Contraindicated	Not contraindicated
LTBI screening	Lower sensitivity with low CD4 T-cell count^20^	Similar sensitivity as in non-DM
TB preventive therapy	Efficacy up to 31%^21^	No data on efficacy
		Not included in guidelines

DM = diabetes mellitus; LAM = lipoarabinomannan; PLWH = people living with HIV; MDR-TB = multidrug-resistant TB; IRIS = immune reconstitution inflammatory syndrome; BCG = bacille Calmette-Guérin; LTBI = latent tuberculous infection.

It is unknown how many people suffer from a combination of TB, HIV and DM. To date, the highest HIV prevalence among TB patients is found in sub-Saharan Africa (SSA), exceeding 50% in parts of southern Africa; the highest reported prevalence rates of DM in TB patients is in India. However, SSA is witnessing the most rapid growth in DM worldwide, while HIV prevalence is rising in other countries, such as Russia, where DM is also common. DM is associated with an estimated 3.5-fold increased risk of TB, with the highest risks found in TB-endemic countries.^23^ Glycaemic control is likely to be an important means of reducing the risk of TB.^24^,^25^

## PATHOGENESIS

HIV-TB pathogenesis is complex, with increased levels of circulating immune complexes found in the early stages and associated with localised tissue necrosis that may lead to increased bacillary numbers.^26^ The excessive type I interferon (IFN) signalling inhibits the immunoprotective effects of type II IFN on *Mtb* infection in macrophages co-infected with HIV-1, leading to inefficient containment of *Mtb* infection and disease reactivation.^27^ HIV-TB coinfection is associated with systemic immune activation,^28^ which precedes CD4 T-cell depletion.^29^ Factors underlying increased susceptibility to TB are still largely unknown in the case of DM, but innate immunity in TB-DM may be underperforming, while adaptive immunity may be exaggerated, with a possible role for excess advanced glycation end products, oxidative stress and epigenetic or immuno-metabolic changes.^30^

### Clinical presentation

Clinical presentation of TB in PLWH varies by CD4 T-cell count: if <350/μL, lung lesions appear similar to those in the non-HIV-infected individuals, including presence of infiltrates in the upper lobe and cavity development. With lower CD4 T-cell counts (e.g., <50/μL), extrapulmonary TB (with potential concomitant pulmonary TB) is more common, accounting for up to 40–80% of the manifestations. Patients may present with lymphadenitis, pleuritis, pericarditis, meningitis, central nervous system tuberculomas, or with disseminated disease manifestations. Radiological images may show atypical features, including lower lobe or middle lobe diseases, miliary infiltrates with lack of cavitations. Transient decrease in CD4 T-cell count and a 5–160-fold rise in HIV load have been demonstrated in TB disease;^31^ this is associated with a more rapid progression of HIV disease.^15^

TB-immune reconstitution inflammatory syndrome (TB-IRIS) is a paradoxical deteriorating or recurring of pre-existing TB lesions, or a development of new lesions in patients on effective anti-TB treatment. In non-HIV-infected patients, prevalence is 2–23%, whereas in PLWH a retrospective meta-analysis of 54 cohorts showed that the incidence of TB-IRIS in patients on ART treatment was 15.7%, with a mortality of 3.2%.^32^ Risk factors for a paradoxical response in PLWH include disseminated disease, lymphopenia or CD4 T-lymphocyte count <50/mm^3^ and a marked increase in lymphocyte count or suppression of HIV-RNA replication with ART. The median time to onset of the paradoxical response is usually 2–4 weeks in PLWH on ART. However, clinicians should be aware that TB-IRIS could occur even later than 4 weeks after anti-TB treatment initiation. Unlike HIV, DM is not associated with extrapulmonary or disseminated TB; however, some studies have shown it is associated with more pulmonary cavities and a higher bacterial load,^16^ Also, diabetic TB patients tend to be older and heavier than TB patients without DM.

### Diagnosis

For the initial pulmonary TB diagnosis, especially in PLWH, a molecular test such as GeneXpert (Cepheid, Sunnyvale, CA, USA) should be used. Microbial diagnosis of HIV-associated TB may be difficult at a late stage of HIV disease due to the (possibly) low number of bacilli detected using standard procedures, and the fact that extrapulmonary TB is more common.^33^ Mycobacterial blood cultures and urine lipoarabinomannan have shown considerable sensitivity in patients with advanced HIV.^14^ In contrast, these tests lack sensitivity in diabetic TB patients and diagnostic approaches are similar to those for patients without DM.

Some studies have suggested a relation between HIV and drug resistance, possibly as a result of nosocomial transmission of drug-resistant strains, but this was not found in a systematic review.^34^ In contrast, two systematic reviews have shown that DM is associated with an almost two-fold higher prevalence of multidrug-resistant TB (MDR-TB), both for primary as well as acquired resistance. One study using whole-genome sequencing has confirmed these findings and shown that mutations associated with resistance to other drugs may also be more common.^35^

### Treatment

TB treatment is similar for both TB-HIV and TB-DM. Although some guidelines have suggested a need for prolonged TB therapy, this is not supported by evidence. TB treatment success rates remain lower among PLWH (77% globally in 2021) than in non-HIV-infected subjects (86% globally in 2021), although the WHO has registered steady improvements over time.^8^ Providing TB treatment and ART to PLWH with TB, is estimated to have averted 74 million deaths between 2000 and 2021.^8^ In PLWH, TB therapy is a major challenge complicated by the optimal timing of ART, drug–drug interactions, overlapping toxicities, IRIS, conditions that may affect treatment adherence and MDR-TB emergence. For drug-susceptible TB, daily administration of isoniazid (INH), rifampicin (RIF), ethambutol, pyrazinamide (PZA) for 2 months is recommended, followed by 4 months of INH and RIF.^36^ A 4-month regimen comprising rifapentine/INH/PZA/moxifloxacin has been shown to be non-inferior to the standard therapy;^8^ however, the trial included only 8% of HIV-infected patients, limiting the comparison of regimens in this population.^37^

ART should be started regardless of CD4 T-cell count within 2 weeks of initiating TB therapy, especially in patients with CD4 <50/mm^3^.^36^ In these patients, early ART initiation significantly reduces TB mortality.^38^ ART should be delayed for at least 4 weeks in PLWH with TB meningitis, so that the risk of severe neurological TB-IRIS may be treated with adjunctive glucocorticoids.^39^ For all forms of drug-resistant TB, the appropriate regimen should be selected on the basis of resistance tests. Recent developments for the treatment of these forms of TB are given in Table 2.^40^ Unless there are drug–drug interaction issues with ART, these regimens can be used regardless of HIV status. A TB-IRIS therapy regimen comprising prednisone treatment during the first 4 weeks after the initiation of ART for HIV infection resulted in a lower incidence of TB-IRIS than placebo, without evidence of an increased risk of severe infections or cancers.^39^,^41^ In TB-DM, TB treatment is similar but therapy failure, disease recurrence, toxicity or dangerous drug interactions are more common.^42^,^43^

**Table 2:** Current indication for drug-resistant TB therapy^40^

Regimen	Indicated to	Comments
6-month BDQ, PMD, LZD (600 mg) and MFX	Patients aged ≥15 years with MDR/RR-TB who have not had previous exposure to BDQ, PMD and LZD (defined as >1 month exposure)	These drugs can be used without MFX in case of documented resistance to FQs
9-month, all-oral, BDQ-containing regimen	Adults and children with MDR/RR-TB, without previous exposure to second-line treatment (including BDQ), without FQ resistance and with no extensive pulmonary TB disease or severe extrapulmonary TB	2 months of LZD (600 mg) can be used as an alternative to 4 months of ethionamide
Individualised longer regimen (18–20 months) designed using resistance test results and priority grouping of medicines recommended in current WHO guidelines	Patients with extensive forms of DR-TB (e.g., XDR-TB or those who are not eligible for or have failed shorter treatment regimens	

BDQ = bedaquiline; PMD = pretomanid; LZD = linezolid; MFX = moxifloxacin; MDR/RR-TB = multidrug-resistant/rifampicin-resistant TB; FQ = fluoroquinolone; DRTB = drug-resistant TB. XDR-TB = extensively drug-resistant TB.

### Drug toxicity

Drug toxicity is a major challenge due to the shared toxicity of ART with anti-TB drugs. Drug-induced liver injury is the most serious adverse event linked to antiretrovirals and some anti-TB drugs such as PZA, RIF and INH.^36^ If drug-induced liver injury is suspected, all potential hepatotoxic drugs should be stopped and administered again when liver function tests have improved.^44^ Cutaneous drug reactions have been reported with thiacetazone, and with nevirapine and abacavir within ART. Severity may range from transient erythematous rash to life-threatening Steven-Johnson syndrome.^44^ Neuro-psychiatric side effects are mainly found with dolutegravir and efavirenz. Among anti-TB drugs, INH, cycloserine and terizidone may cause psychosis and suicidal ideation.^44^ Prolongation of the QT interval is associated with several TB drugs (fluoroquinolones, bedaquiline [BDQ], clofazimine), and require close electrocardiogram monitoring during treatment.^44^ In TB-DM patients who are on average 10–15 years older than TB-only patients, liver fibrosis, diabetic nephropathy and concomitant drug treatment may lead to more drug toxicity and side effects.

### Drug interactions

Important agents in TB therapy with high sterilising activity, rifamycins are responsible for several drug interactions as they induce cytocrome P450 enzyme-3A4 and uridine disphosphate glucoronosyltrasferase-1A1 and are P-glycoprotein transporters.^44^ The most significant RIF drug–drug interactions with ART are shown in Table 3.^45^,^46^ RIF substantially lowers levels of most oral drugs, but not metformin,^47^ which likely improves TB treatment outcomes. Insulin is often advocated as first choice in TB-DM as it is not metabolised, but it may be inaccessible or hard to use in low-resource settings. Limited data are available on interactions between ART and drugs for RIF-resistant TB. BDQ is a CYP3A4 substrate, thus drugs that inhibit this enzyme (such as ritonavir-boosted PIs), may lead to an increase in BDQ concentration, increasing its toxicity as QT-prolongation. In contrast, nevirapine and rilpivirine do not appear to have a significant effect on BDQ exposure. For delamanid and linezolid, interactions with ART are considered unlikely.^48^

**Table 3:** Interactions of RIF with HIV and DM drugs

	RIF	Notes	Source
HIV			
NRTI	↓ or ↑	No interaction expected except for TAF. Co-administration of RIF and TAF results in reduction of TDF DP in plasma and cells; however, pharmacokinetic studies showed that intracellular TDF DP concentrations were still 4.21-fold higher than TDF disoproxil-fumarate	44
NNRTI	↓	Plasma concentrations of NVP, rilpivirine, etravirine and dorvirine are significantly affected and these drugs should be avoided.^41^ Doravirine exposure is reduced by 50% with concomitant RBT, but a twice-daily dose appears to overcome induction^42^	44,45
INI	↓	Interaction of RIF with the INI raltegravir and dolutegravir can be overcome by a twice-daily dose of the drugs. The use of elvitegravir/cobicistat and bictegravir is contraindicated as they are CYP3A4 and UGT1A1 substrates. Also, the combination of RIF with long-acting rilpivirine-cabotegravir could lead to subtherapeutic concentrations and so should be avoided^41^	44
PI	↓	PI should be avoided with RIF as the latter reduces PI plasma concentrations. RBT can substitute for RIF when the use of PI is essential, as it induces CYP3A4 to a lesser extent; however, the RBT dose must be reduced to 150 mg when administered concomitantly with PI-boosters to avoid an increase in RBT concentrations. The use of cobicistat should be avoided^41^	44
DM			
Insulin	No interaction	Not metabolised by cytochromes induced by RIF, but some limitations in its use: need of self-monitoring, risk of hypoglycaemia, may be inaccessible or hard to use in low-resource settings	46
Metformin	Generally ↑	First choice in TB patients. RIF may increase the effects of metformin but generally with limited clinical effect. Additional monitoring of dose adjustment may be necessary	46
Sulphonylurea derivates	↓ or ↑	RIF strongly lowers levels of most oral drugs. Used in case there is a contraindication or intolerance to metformin	47

RIF = rifampicin; DM = diabetes mellitus; NRTI = nucleoside/nucleotide reverse transcriptase inhibitors; TAF = TDF-alafenamide; TDF = tenofovir; DP = diphosphate; NNRTI = non-nucleoside/nucleotide reverse transcriptase inhibitors; NVP = nevirapine; RBT = rifabutin; INI = integrase inhibitors; PI = protease inhibitors.

### Prevention and control

Prevention of HIV-associated TB now largely relies on TB disease screening and TB preventive therapy (TPT).^28^ These measures contribute to reducing the risk of TB reactivation in PLWH with TB infection (TBI).^49^ Current tests for TBI have a lower sensitivity in people with advanced HIV,^20^ and according to the WHO, all HIV-infected individuals should receive TPT irrespective of the TBI test result.^3^ However, the yield of TBI screening and TPT in low TB-endemic settings is currently unknown, and some guidelines in Western countries now advocate a more risk-stratified approach. There are several regimes for TPT, including a 1-month regimen of combined daily rifapentine and INH that proved effective in HIV-infected people.^28^

Current guidelines do not recommend TPT for DM patients, but one study in Indonesia has shown a high incidence among them and a positive interferon-gamma release assay result;^50^ the first randomised clinical trial examining the effect and safety of TPT among diabetics has started in Uganda and Tanzania (Clinical Trial Registration NCT04600167).^51^ Other proposed measures to control TB-HIV and TB-DM include a more effective vaccine than bacille Calmette-Guérin, which mainly protects against childhood TB, along with better control of both HIV and DM.

## CONCLUSION

Both HIV infection and DM have a strong impact on the clinical presentation, diagnosis and management of TB. It is vital that we strengthen our efforts to prevent these comorbidities from occurring, and implement screening, early diagnosis and appropriate management strategies to reduce the burden of TB disease.

## References

[1] Migliori GB (2021). Gauging the impact of the COVID-19 pandemic on tuberculosis services: a global study. Eur Respir J.

[2] Petruccioli E (2016). Correlates of tuberculosis risk: predictive biomarkers for progression to active tuberculosis. Eur Respir J.

[3] World Health Organization (2020). WHO consolidated guidelines on tuberculosis: tuberculosis preventive treatment. Module 1: prevention.

[4] World Health Organization (2021). Global Diabetes Summit.

[5] World Health Organization (2022). WHO HIV report, 2022.

[6] Noubiap JJ (2019). Global prevalence of diabetes in active tuberculosis: a systematic review and meta-analysis of data from 2·3 million patients with tuberculosis. Lancet Glob Health.

[7] International Diabetes Federation (2021). IDF Diabetes Atlas.

[8] World Health Organization (2022). Global tuberculosis report, 2022.

[9] World Health Organization (2021). TB & diabetes.

[10] Liu Q (2022). The association between diabetes mellitus and the risk of latent tuberculosis infection: a systematic review and meta-analysis. Front Med (Lausanne).

[11] Ellis PK, Martin WJ, Dodd PJ (2017). CD4 count and tuberculosis risk in HIV-positive adults not on ART: a systematic review and meta-analysis. Peer J.

[12] Foe-Essomba JR (2021). Diabetes mellitus and tuberculosis, a systematic review and meta-analysis with sensitivity analysis for studies comparable for confounders. PLoS One.

[13] Al-Rifai RH (2017). Association between diabetes mellitus and active tuberculosis: a systematic review and meta-analysis. PLoS One.

[14] Lawn SD (2015). Rapid microbiological screening for tuberculosis in HIV-positive patients on the first day of acute hospital admission by systematic testing of urine samples using Xpert MTB/RIF:a prospective cohort in South Africa. BMC Med.

[15] Munsiff SS (1998). A prospective study of tuberculosis and HIV disease progression. J Acquir Immune Defic Syndr Hum Retrovirol.

[16] Tong X (2021). Clinical features in pulmonary tuberculosis patients combined with diabetes mellitus in China: an observational study. Clin Respir J.

[17] Sultana ZZ (2021). HIV infection and multidrug resistant tuberculosis: a systematic review and meta-analysis. BMC Infect Dis.

[18] Tegegne BS (2018). Association between diabetes mellitus and multi-drug-resistant tuberculosis: evidence from a systematic review and meta-analysis. Syst Rev.

[19] Huangfu P (2019). The effects of diabetes on tuberculosis treatment outcomes: an updated systematic review and meta-analysis. Int J Tuberc Lung Dis.

[20] Petruccioli E (2020). Effect of HIV-infection on QuantiFERON-plus accuracy in patients with active tuberculosis and latent infection. J Infect.

[21] Jagi JL Efficacy, safety, and tolerability of isoniazid preventive therapy for tuberculosis in people living with HIV:a systematic review and meta-analysis. AIDS.

[22] Gupta A (2012). Tuberculosis incidence rates during 8 years of follow-up of an antiretroviral treatment cohort in South Africa: comparison with rates in the community. PLoS One.

[23] Baker MA (2011). The impact of diabetes on tuberculosis treatment outcomes: a systematic review. BMC Med.

[24] Lee P-H (2016). Glycemic control and the risk of tuberculosis: a cohort study. PLoS Med.

[25] Shewade HD (2017). Effect of glycemic control and type of diabetes treatment on unsuccessful TB treatment outcomes among people with TB-diabetes: a systematic review. PLoS One.

[26] Esmail H (2018). Complement pathway gene activation and rising circulating immune complexes characterize early disease in HIV-associated tuberculosis. Proc Natl Acad Sci USA.

[27] Moreira-Teixeira L (2018). Type I interferons in tuberculosis: foe and occasionally friend. J Exp Med.

[28] Goletti D (2022). The role of IGRA in the diagnosis of tuberculosis infection, differentiating from active tuberculosis, and decision making for initiating treatment or preventive therapy of tuberculosis infection. Int J Infect Dis.

[29] Geldmacher C (2008). Early depletion of *Mycobacterium tuberculosis*specific T helper 1 cell responses after HIV-1 infection. J Infect Dis.

[30] Ronacher K (2017). Defining a research agenda to address the converging epidemics of tuberculosis and diabetes. Part 2: Underlying biologic mechanisms. Chest.

[31] Goletti D (1996). Effect of *Mycobacterium tuberculosis* on HIV replication. Role of immune activation. J Immunol.

[32] Müller M (2010). Immune reconstitution inflammatory syndrome in patients starting antiretroviral therapy for HIV infection: a systematic review and meta-analysis. Lancet Infect Dis.

[33] Lawn SD (2012). Diagnostic accuracy of a low-cost, urine antigen, point-of-care screening assay for HIV-associated pulmonary tuberculosis before antiretroviral therapy: a descriptive study. Lancet Infect Dis.

[34] Gandhi NR (2013). Nosocomial transmission of extensively drug-resistant tuberculosis in a rural hospital in South Africa. J Infect Dis.

[35] Ruesen C (2020). Diabetes is associated with genotypically drug-resistant tuberculosis. Eur Respir J.

[36] World Health Organization (2022). WHO consolidated guidelines on tuberculosis. Module 4: Treatment: drug-susceptible tuberculosis treatment.

[37] Dorman SE (2021). Four-month rifapentine regimens with or without moxifloxacin for tuberculosis. N Engl J Med.

[38] Uthman OA (2015). Optimal timing of antiretroviral therapy initiation for HIV-infected adults with newly diagnosed pulmonary tuberculosis: a systematic review and meta-analysis. Ann Intern Med.

[39] Meintjes G (2018). Prednisone for the prevention of paradoxical tuberculosis-associated IRIS. N Engl J Med.

[40] World Health Organization (2022). Rapid communication: key changes to the treatment of drug-resistant tuberculosis.

[41] Meintjes G (2010). Randomized placebo-controlled trial of prednisone for paradoxical tuberculosis-associated immune reconstitution inflammatory syndrome. AIDS.

[42] Alkabab Y (2023). Therapeutic drug monitoring and TB treatment outcomes in patients with diabetes mellitus. Int J Tuberc Lung Dis.

[43] Forsman L (2023). Diabetes mellitus and TB – finding strategies to reduce the double burden of disease. Int J Tuberc Lung Dis.

[44] Cerrone M (2019). Rifampicin effect on intracellular and plasma pharmacokinetics of tenofovir alafenamide. J Antimicrob Chemother.

[45] Khalilieh SG (2018). Multiple doses of rifabutin reduce exposure of doravirine in healthy subjects. J Clin Pharmacol.

[46] Drugs.com Drug interactions checker: for drugs, food & alcohol. https://www.drugs.com/drug_interactions.html.

[47] Te Brake LHM (2019). Rifampicin alters metformin plasma exposure but not blood glucose levels in diabetic tuberculosis patients. Clin Pharmacol Ther.

[48] Hamada Y (2021). HIV-associated tuberculosis. Int J STD AIDS.

[49] Campbell J R (2021). Safety and efficacy of rifampin or isoniazid among people with *Mycobacterium tuberculosis* infection and living with human immunodeficiency virus or other health conditions: post hoc analysis of 2 randomized trials. Clin Infect Dis.

[50] Koesoemadinata RC (2017). Latent TB infection and pulmonary TB disease among patients with diabetes mellitus in Bandung, Indonesia. Trans R Soc Trop Med Hyg.

[51] Ntinginya NE (2022). Rifapentine and isoniazid for prevention of tuberculosis in people with diabetes (PROTID): protocol for a randomised controlled trial. Trials.

